# Quality of reporting according to the CONSORT, STROBE and Timmer instrument at the American Burn Association (ABA) annual meetings 2000 and 2008

**DOI:** 10.1186/1471-2288-11-161

**Published:** 2011-11-29

**Authors:** Karsten Knobloch, Uzung Yoon, Hans O Rennekampff, Peter M Vogt

**Affiliations:** 1 Plastic, Hand and Reconstructive Surgery, Burn Center, Hannover Medical School, Carl-Neuberg-Str. 1, Hannover, 30625, Germany; 2 Department of Surgery, New York Hospital Queens, 56-45 Main Street, Flushing, N.Y. 11355, USA

**Keywords:** Burns, evidence, consort, strobe, timmer, reporting quality, abstract

## Abstract

**Background:**

The quality of oral and poster conference presentations differ. We hypothesized that the quality of reporting is better in oral abstracts than in poster abstracts at the American Burn Association (ABA) conference meeting.

**Methods:**

All 511 abstracts (2000: N = 259, 2008: N = 252) from the ABA annual meetings in year 2000 and 2008 were screened. RCT's and obervational studies were analyzed by two independent examiners regarding study design and quality of reporting for randomized-controlled trials (RCT) by CONSORT criteria, observational studies by the STROBE criteria and additionally the Timmer instrument.

**Results:**

Overall, 13 RCT's in 2000 and 9 in 2008, 77 observational studies in 2000 and 98 in 2008 were identified. Of the presented abstracts, 5% (oral; 7%(n = 9) vs. poster; 3%(n = 4)) in 2000 and 4% ((oral; 5%(n = 7) vs. poster; 2%(n = 2)) in 2008 were randomized controlled trials. The amount of observational studies as well as experimental studies accepted for presentation was not significantly different between oral and poster in both years. Reporting quality of RCT was for oral vs. poster abstracts in 2000 (CONSORT; 7.2 ± 0.8 vs. 7 ± 0, p = 0.615, CI -0.72 to 1.16, Timmer; 7.8 ± 0.7 vs. 7.5 ± 0.6,) and 2008 (CONSORT; 7.2 ± 1.4 vs. 6.5 ± 1, Timmer; 9.7 ± 1.1 vs. 9.5 ± 0.7). While in 2000, oral and poster abstracts of observational studies were not significantly different for reporting quality according to STROBE (STROBE; 8.3 ± 1.7 vs. 8.9 ± 1.6, p = 0.977, CI -37.3 to 36.3, Timmer; 8.6 ± 1.5 vs. 8.5 ± 1.4, p = 0.712, CI -0.44 to 0.64), in 2008 oral observational abstracts were significantly better than posters (STROBE score; 9.4 ± 1.9 vs. 8.5 ± 2, p = 0.005, CI 0.28 to 1.54, Timmer; 9.4 ± 1.4 vs. 8.6 ± 1.7, p = 0.013, CI 0.32 to 1.28).

**Conclusions:**

Poster abstract reporting quality at the American Burn Association annual meetings in 2000 and 2008 is not necessarily inferior to oral abstracts as far as study design and reporting quality of clinical trials are concerned. The primary hypothesis has to be rejected. However, endorsement for the comprehensive use of the CONSORT and STROBE criteria might further increase the quality of reporting ABA conference abstracts in the future.

## Background

Regularly physicians and experts of scientific research meet in conference to publish their results and to share information. Mostly, the results are presented in an abstract form. An abstract should at best provide the reader with an efficient summary of the study that facilitates scanning many articles to find those that are the most pertinent to the reader's interests and needs [1]. Furthermore, abstracts from expert meetings like annual meeting of American Burn Association (ABA) are likely to influence clinical decisions. Therefore, the role and importance of an abstract is not to be underestimated. Generally, after an abstract is submitted for a conference will undergo peer-review. Issues like topic and conference arrangement are likely to influence the decision whether a abstract will be presented as poster or oral.

Within the last decade several efforts have been undertaken to improve reporting quality of abstracts according to evidence-based medicine (EBM) criteria. The publication of the CONSORT Statement in 1996 was an important step toward improving reporting standards. In 1996, a group from the Department of Epidemiology and Biostatistics of the Memorial Sloan Kettering Cancer Center in New York City published their first report entitled: "Improving the quality of reporting of randomized controlled trials, the CONSORT statement [2]. This was designed as a guideline to improve quality in reporting RCTs by using a checklist and a flow diagram [3]. Some of the items in the checklist include enrollment, intervention allocation, follow-up, and analysis. Recently the CONSORT group has adapted their recommendations to journal and conference abstracts on RCT's [4]. The conference abstracts checklist is strongly recommended by the CONSORT group to increase clarification.

Somewhat similar to the aforementioned CONSORT criteria for randomized-controlled trials, the STROBE statement has been instituted for observational studies in order to improve the reporting quality of case-control and cohort studies. A checklist of 22 items was presented addressing three main study designs of analytical epidemiology: cohort, case-control, and cross-sectional studies. Twenty-three individuals attended a meeting in September 2004, Bristol, UK, including editorial staff from *Annals of Internal Medicine, BMJ, Bulletin of the World Health Organization, International Journal of Epidemiology, JAMA, Preventive Medicine, and The Lancet*, epidemiologists, methodologists, statisticians, and practitioners from Europe and North America. They identified items deemed to be important to include in checklists for each type of study and decided on the strategy for finalizing and disseminating the STROBE Statement [5].

Timmer et al. noted the lack of an abstract quality evaluating instrument and the need to improvement of abstract quality. In 2003, they developed a reliable and valid instrument to assess the quality of meeting abstracts that would be applicable to a wide variety of research types, including both clinical and basic science [6]. One of the benefits is Timmer instrument is applicable for all study designs from metanalyses, RCTs, observational studies, case studies to experimental studies.

Often times poster abstracts presented at conference meetings are perceived to be somewhat inferior for yet unknown reasons. The aim of this study was to determine the abstract reporting quality in randomized controlled trials and observational studies applying the CONSORT, STROBE checklist and Timmer instrument. We hypothesized that poster presented abstracts would have an inferior reporting quality score than orally presented abstracts.

## Methods

### Inclusion criteria

A total number of 511 abstracts presented at the American Burn Association annual meeting in 2000 (259 abstracts) and 2008 (252 abstracts) were screened according to the quality of reporting. According to evidence-based medicine, the study design (randomized controlled trial, observational study, case series, experimental study) was determined. Clinical studies with the known study design according to the level of evidence were discriminated from experimental studies. In abstracts without mentioned study design the study design was evaluated based on the method part, prospective, retrospective, follow-up method, intervention, data gathering method and description of the results.

A RCT was defined if the abstract described a prospective study in which individuals are allocated at random to an intervention or to a controll group. A porspective or retrospective study that involves identification of two groups (cohorts) of patients, one which did receive the exposure of interest, and one which did not, and following these cohorts forward for the outcome of interest wa s defined as cohort study. A retrospective study that compared the exposure of individual in two groups, one of which had the condition under consideration and one which did not" was definined of a case control study. Video studies was defined as those which descibed a evaluation of a video. Case report was defined as reporting of an individual or on a series of patients with no control group, intervention or statistical analysis. Systematical review was defined as a literature review focused on a single question which tries to identify, appraise, select and synthesis all high quality research evidence relevant to that question. If the study describes purely about a surgery technique without control group it was defined as a describing op technique. If the study was conducted with any kind of animals, tissue or cells it was defined as an experimental study. In the study desing was still unlcer or did not match with the definition above, abstracts were re-analysed by the researchers (UY and KK) together. The type and method of intervention was discussed and it was searched for words like placebo, blinded, random, placebo, questionare, lab techniques (PCR, western/southern/northern-blot), odds ratio, relative risk to analyse the study design.

For each RCT or observational study conference abstract, the score for CONSORT abstract or STROBE was calculated by two independent researchers (UY and KK). The scores of each abstract were compared and the mean were used when the data varies. Additionally the timmer instrument was calculated for each abstract and by inconsistencis again the mean was used. Experimental studies were not analysed for reporting quality.

Both researchers were blinded for journal year, oral/poster, author and institution. The mean score of both investigators was taken. Interpreter variability was calculated by t-test (p = 0.873) and Pearson correlation (0.761).

CONSORT criteria for abstract [7] (additional file 1) were applied for randomised-controlled trial abstracts and STROBE criteria (additional file 2) for observational studies. Scores were calculated for 17 CONSORT and 22 STROBE criteria. One point was given if the abstract meets the items proposed in the criteria and zero points if not met (CONSORT score range: 0-17, STROBE score range: 0-22). All individual criteria were weighted equally.

In addition to the CONSORT and STROBE score the Timmer quality scoring instrument (additional file 3) was applied for all abstracts. The Timmer instrument consists of 19 items as a quality index which was developed for the evaluation of scientific meeting abstracts in a standardized way. We modified the score calculation to focus on the reporting quality of the abstracts. Therefore we excluded the study design in the scoring system and 0-1 points were given for each item (1 if met, 0 if not met). For the item which is not applicable, such as controll group (item 5), randomisation (item 7) or blinding (item 8, 9) of subjects in observational studies, the points were subtracted from the score. Therefore for randomized controlled trials the score range was 0-19 points and for observational studies 0-15 points.

### Outcome measures

The study design (randomized controlled trial, non-randomized trial, experimental, prospective, retrospective), 17 CONSORT and 22 STROBE criterias for reporting in conference abstracts, and the Timmer instrument for abstract quality were determined.

### Statistics

Descriptive statistics consisted of the calculation of frequencies and percentages. T-test was used to calculate and compare oral vs poster according to CONSORT and STROBE score in 2000 and 2008 respectively. The Chi-square tests were applied for assessment of the improvement between each CONSORT and STROBE criteria in 2000 and 2008. Fisher exact tests were used in the analysis of categorical data where sample sizes were small. Data were analyzed using the SPSS statistical software package Version 14.0.

## Results

### Evidence-based study design of American Burn Association annual meeting abstracts

Overall, 511 accepted and presented abstracts (2000: N = 259, 2008: N = 252) were screened. 13 RCT's in 2000 and 9 in 2008, 77 observational studies in 2000 and 98 in 2008 were included for analysis. Of all the presented abstracts from the annual meeting, 5% (oral; 7%(n = 9) vs. poster; 3%(n = 4)) in 2000 and 4% ((oral; 5%(n = 7) vs. poster; 2%(n = 2)) in 2008 were randomized controlled trials. There were no significant differences in the amount of presented RCTs between oral and poster (2000; p = 0.268 vs. 2008; p = 0.325). Overall, *ABA *conference abstracts, 48% were (oral; 56% (n = 77) vs. poster; 39% (n = 47)) observational studies in 2000 and 64% (oral; 65% (n = 98) vs. poster; 64% (n = 64)) in 2008. Significantly more observational studies were presented in 2000 annual meeting (2000; p = 0.006 vs. 2008; p = 0.939). Experimental studies were presented in 32% (oral; 30% (n = 42) vs. poster; 32% (n = 39), p = 0.478) and 29% (oral; 29% (n = 244) vs. poster; 30% (n = 30), p = 0.727) respectively with no statistical differences (table 1).

**Table 1:** Study type of the presented abstract at the BURNS annual meeting 2000 and 2008

	2000					2008				
	**oral**	**%**	**poster**	**%**	**p**	**oral**	**%**	**poster**	**%**	**p**

**RCT**	9	7	4	3	0.268	7	5	2	2	0.325

**non RCT**	87	63	74	61	0.755	101	66	68	68	0.927

Observational	*77*	*56*	*47*	*39*	0.006*	*98*	*64*	*64*	*64*	*0.939*

Other (Video, Case report, Systematical Review, Describing op/new technik)	*10*	*7*	*27*	*22*	< 0.001*	*3*	*2*	*4*	*4*	-

**Experimental**	42	30	39	32	0.478	44	29	30	30	0.727

**Drop Out**	0	0	4	3	0.046*	-	-	-	-	-

**Total**	**138**		**121**			**152**		**100**		

RCT: Randomized-controlled trial, Non-RCT: non-randomized controlled trial

* Statistically significant (p < 0.05)

Drop out: These abstracts were marked in the burns annual meeting abstract-book as "drop out" and were deleted.

### CONSORT criteria and Timmer quality instrument for reporting randomised-controlled trials in annual meeting abstracts

**Figure 1:**
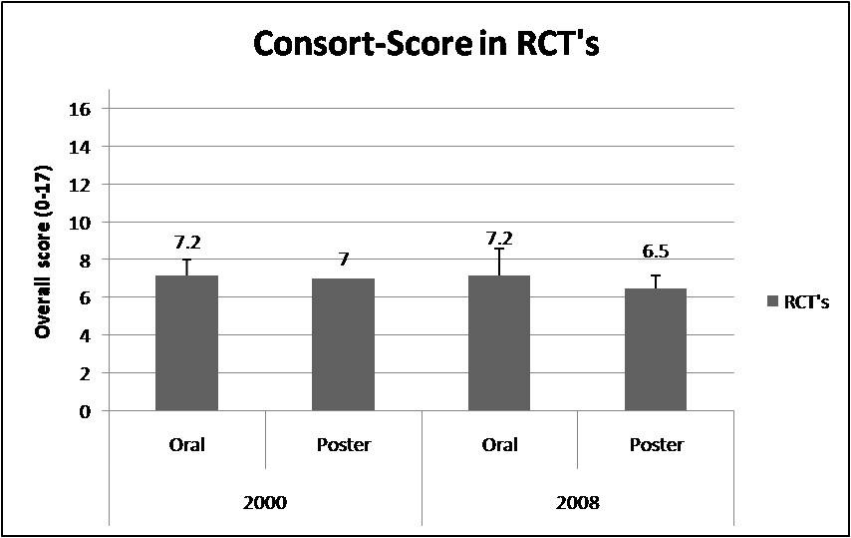
**Consort-Score in RCT's**. Consort-score in RCT's- A comarison between oral and poster presentation in 2000 and 2008.

**Figure 2:**
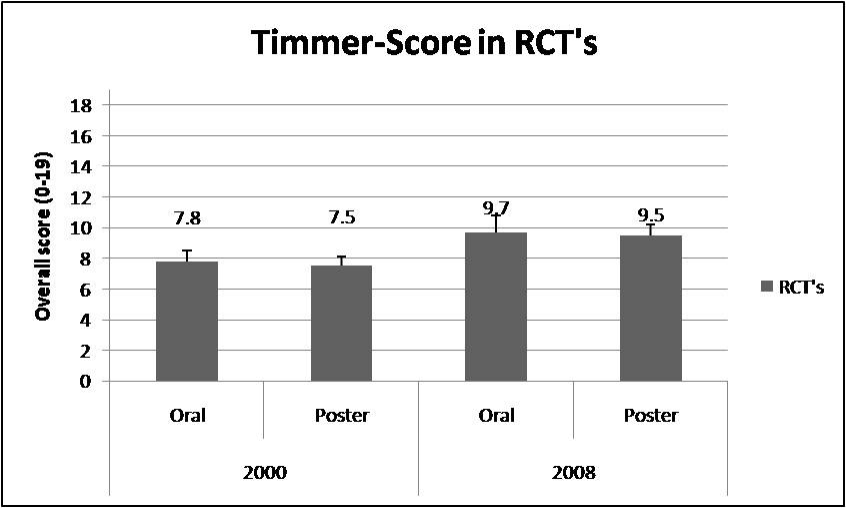
**Timmer-Score in RCT's**. Timmer-score in RCT's-A comarison between oral and poster presentation in 2000 and 2008.

Reporting quality of RCT was for oral vs. poster abstracts in 2000 (CONSORT; oral: 7.2 ± 0.8 vs. poster: 7 ± 0) and 2008 (CONSORT; oral: 7.2 ± 1.4 vs. poster: 6.5 ± 1, Timmer; 9.7 ± 1.1 vs. 9.5 ± 0.7) Figure 1). The Timmer instrument for RCTs in *ABA *2000 meeting were 7.8 ± 0.7 (oral) and 7.5 ± 0.6 (poster,), respectively. In 2008 the Timmer instrument were 9.7 ± 1.1 for oral and 9.5 ± 0.7 for poster presentations (p = 0.819, CI -1.79 to 2.19, Figure 2). There were no significant reporting quality differences between oral and poster presented abstracts. Details of numbers and percentages for each item are shown on table 2, 3.

**Table 2:** Quality of reporting of abstracts at BURNS annual meeting 2000 and 2008 according to the CONSORT criteria for randomized-control trials

CONSORT	2000				2008			
	**oral**		**poster**		**oral**	**poster**		

	**N**	**%**	**N**	**%**	**N**	**%**	**N**	**%**

**Total**	9	100	4	100	7	100	2	100

**Title randomized**	0	0	1	25	1	14	1	50

**Authors**	0	0	0	0	0	0	0	0

**Trial design**	9	100	2	50	6	86	1	50

**Participants**	8	89	4	100	4	57	0	0

**Interventions**	9	100	4	100	6	86	2	100

**Objective/Hypothesis**	9	100	4	100	6	86	2	100

**Main Outcome**	2	22	0	0	1	14	1	50

**Randomization**	0	0	0	0	1	14	0	0

**Blinding**	2	22	0	0	4	57	0	0

**Number randomized**	0	0	1	25	0	0	0	0

**Recruitment**	0	0	0	0	0	0	0	0

**Numbers analyzed**	8	89	4	100	3	43	2	100

**Outcome**	9	100	4	100	7	100	2	100

**Harms**	0	0	0	0	1	14	0	0

**Conclusion**	9	100	4	100	7	100	2	100

**Trial registration**	0	0	0	0	0	0	0	0

**Funding**	0	0	0	0	3	43	0	0

Due to the small sample size p-value was not calculated.

**Table 3:** Quality of reporting of abstracts at BURNS annual meeting 2000 and 2008 according to the Timmer instrument for randomized-control trials

Timmer instrument for RCTs	2000				2008			
	**oral**		**poster**		**oral**		**Poster**	

	**N**	**%**	**n**	**%**	**n**	**%**	**n**	**%**

Question/objective sufficiently described?	5	56	4	100	7	100	2	100

Design evident and appropriate to answer study question?	8	89	3	75	6	86	2	100

Subject characteristics sufficiently described?	7	78	0	0	5	71	2	100

Subjects appropriate to the study question?	7	78	3	75	6	86	1	50

Controls used and appropriate? **(if no control, check no)**	9	100	4	100	7	100	2	100

Method of subject selection described and appropriate?	0	0	0	0	2	29	0	0

If random allocation to treatment groups was possible, is it described? (if not possible, check n/a)	0	0	1	25	1	14	0	0

If blinding of investigators to intervention was possible, is it reported? (If not possible, n/a)	0	0	0	0	1	14	0	0

If blinding of subjects to intervention was possible, is it reported? (If not possible, n/a)^1^	2	22	0	0	3	43	0	0

Outcome measure well defined and robust to measurement bias? Means of assessment reported?	7	78	4	100	5	71	2	100

Confounding accounted for?	0	0	0	0	0	0	0	0

Sample size adequate?	0	0	0	0	0	0	0	0

Post hoc power calculations or confidence intervals reported for statistically non significant results?	0	0	0	0	0	0	0	0

Statistical analyses appropriate?	2	22	1	25	3	43	1	50

Statistical tests stated?	0	0	1	25	1	14	1	50

Exact p-values or confidence intervals stated?	7	78	2	50	7	100	2	100

Attrition of subjects and reason for attrition recorded?	0	0	0	0	0	0	0	0

Results reported in sufficient detail?	7	78	3	75	7	100	2	100

Do the results support the conclusions?	9	100	4	100	7	100	2	100

Sum (items 1-19)								

### STROBE criteria and Timmer quality instrument for reporting observational studies in annual meeting abstracts

**Figure 3:**
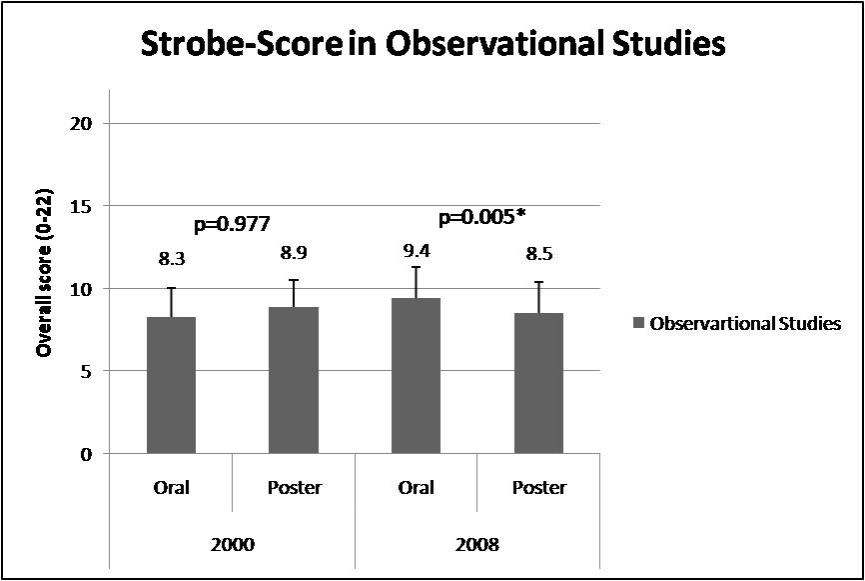
**Strobe-Score in Observational Studies**. Strobe-score in observational studies-A comarison between oral and poster presentation in 2000 and 2008.

**Figure 4:**
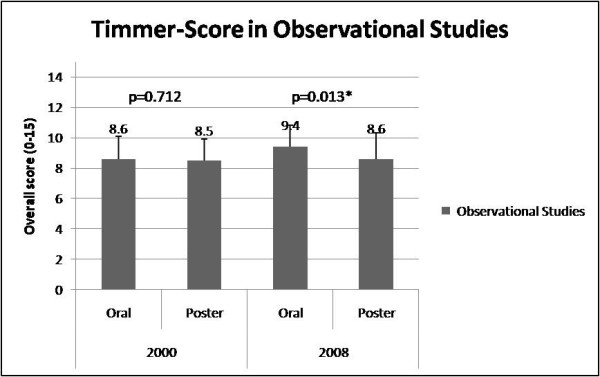
**Timmer-Score in Observational Studies**. Timmer-score in observational studies-A comarison between oral and poster presentation in 2000 and 2008.

According to STROBE criteria, the 2000 annual meeting displayed no significant differences in reporting quality of observational studies in oral and poster presentations [oral: 8.3 ± 1.7 (score range: 4-12) vs. poster: 8.9 ± 1.6 (score range: 5-12), p = 0.977, CI -37.3 to 36.3]. Likewise, the Timmer instrument revealed no significant differences for oral: [8.6 ± 1.5 (score range: 6-12)] vs. poster abstracts [8.5 ± 1.4 (score range: 5-12), p = 0.712, CI -0.44 to 0.64]. In 2008, oral presented observational abstracts had a significantly better reporting quality than poster presented abstracts [oral: 9.4 ± 1.9 (score range: 4-13), vs. poster: 8.5 ± 2 (score range: 5-14), p = 0.005, CI 0.28 to 1.54, Figure 3]. Also the Timmer instrument was significantly better for oral vs. poster observational studies in 2008 (oral: 9.4 ± 1.4 (score range: 5-12), vs. poster: 8.6 ± 1.7 (score range: 6-11), p = 0.013, CI 0.32 to 1.28, Figure 4).

The significant differences in 2008 oral and poster reporting quality in observational studies occured in the sections objectives (74%(n = 73) vs. 39%(n = 25), p < 0.001), data sources (32%(n = 31) vs. 17%(n = 11), p = 0.037), study size (12% (n = 12) vs. 0%(n = 0), p = 0.004), quantitative variable (12%(n = 12) vs. 2%(n = 1), p = 0.016), and interpretation (87%(n = 85) vs. 100%(n = 64, p = 0.004, table 4, 5)

**Table 4:** Quality of reporting of abstracts at BURNS annual meeting 2000 and 2008 according to the STROBE criteria for observational studies

STROBE	2000					2008				
	**oral**		**poster**			**oral**		**poster**		

	**N**	**%**	**n**	**%**	**p-value**	**n**	**%**	**n**	**%**	**p-value**

**Total**	77	100	47	100		98	100	64	100	

**Title and abstract**	0	0	0	0	-	2	2	1	2	1

**Background/rationale**	72	94	46	98	0.407	88	90	64	100	0.012

**Objectives**	50	65	36	77	0.172	73	74	25	39	< 0.001*

**Study design**	15	19	12	26	0.428	21	21	16	25	0.621

**Setting**	27	35	23	49	0.127	46	47	37	58	0.197

**Participants**	44	57	27	57	0.974	42	43	21	33	0.182

**Variables**	51	66	29	62	0.609	47	48	37	58	0.245

**Data sources/measurement**	25	32	18	38	0.508	31	32	11	17	0.037*

**Bias**	0	0	1	2	0.379	0	0	0	0	-

**Study size**	0	0	0	0	-	12	12	0	0	0.004*

**Quantitative variables**	9	12	3	6	0.533	12	12	1	2	0.016*

**Statistical methods**	15	19	7	15	0,512	27	28	10	16	0.072

**Participants**	30	39	24	51	0.187	56	57	41	64	0.422

**Descriptive data**	9	12	15	32	0.006*	31	32	23	36	0.601

**Outcome data**	61	79	43	91	0.083	81	83	58	91	0.198

**Main result**	66	86	44	94	0.246	81	83	60	94	0.085

**Other analysis**	0	0	0	0	-	0	0	0	0	-

**Key result**	63	82	43	91	0.19	83	85	61	95	0.066

**Limitation**	0	0	1	2	0.379	0	0	0	0	-

**Interpretation**	72	94	45	96	0.708	85	87	64	100	0.004*

**Generalizability**	0	0	0	0	-	0	0	0	0	-

**Funding**	0	0	0	0	-	24	24	15	23	0.85

* Statistically significant (p < 0.05)

**Table 5:** Quality of reporting of abstracts at BURNS annual meeting 2000 and 2008 according to the STROBE criteria for observational studies

Timmer instrument for observational studies	2000				2008			
	**oral**		**poster**		**0ral**		**poster**	

	**n**	**%**	**n**	**%**	**n**	**%**	**n**	**%**

Question/objective sufficiently described?	50	65	36	77	73	74	25	63

Design evident and appropriate to answer study question?	75	97	46	98	95	97	61	95

Subject characteristics sufficiently described?	45	58	27	57	61	62	36	56

Subjects appropriate to the study question?	68	88	41	87	95	97	54	84

Method of subject selection described and appropriate?	61	79	44	94	81	83	52	81

Outcome measure well defined and robust to measurement bias? Means of assessment reported?	25	32	18	38	73	74	37	58

Confounding accounted for?	0	0	1	2	0	0	0	0

Sample size adequate?	0	0	0	0	12	12	0	0

Post hoc power calculations or confidence intervals reported for statistically non significant results?	0	0	0	0	0	0	0	0

Statistical analyses appropriate?	71	92	35	74	89	91	57	89

Statistical tests stated?	15	19	7	15	27	28	10	16

Exact p-values or confidence intervals stated?	72	92	40	85	90	92	58	88

Attrition of subjects and reason for attrition recorded?	34	44	20	43	54	55	42	66

Results reported in sufficient detail?	74	96	40	85	82	84	61	98

Do the results support the conclusions?	72	94	45	96	89	91	56	88

Sum (items 1-15)								

## Discussion

The major findings of this work are: Poster abstract reporting quality at the American Burn Association annual meeting of 2000 and 2008 is not necessarily inferior to oral abstracts as far as study design and reporting quality of clinical trials is concerned. The primary hypothesis has to be rejected. However, endorsement for the comprehensive use of the CONSORT and STROBE criteria might further increase the quality of reporting ABA conference abstracts in the future.

These observations should be discussed in detail. This is the first reporting quality evaluation and comparing study in the field of burn surgery. Evidence-based criteria such as study design and measure of reporting quality like the CONSORT and the STROBE score are useful for abstractcomparison. The increased assessment of study quality has led to the development of numerous scales and checklists [8]. In the last years, CONSORT and STROBE checklists were used more frequently to analyse the reporting quality in published studies [9,10]. Additionally, calculating a scale provides readers a quantitative index of trial quality by assigning a numeric scores to each item present, contributing to an overall total.

In the *ABA *annual meeting the oral and poster abstract reporting quality revealed no differences, but the number of presented RCTs was still very limited with 5% at best. These results are consistent with other studies especially in the surgical field [11,12]. A tenyear analysis of publications in *Plastic and Reconstructive Surgery, Annals of Plastic Surgery and Aesthetic Surgery Journal *revealed a RCT publication rate of 3.2% [13]. Major reasons for the lack of RCTs are the complexity of study design associated with intensive patient care, expenditure of time and high costs. And also, ethical considerations in interventions are a challange in realizing RCTs. However, a surgical RCT enables optimal properties to minimize bias in the estimation of the treatment effect which can otherwise easily be underestimated or exaggerated. RCTs are widely acknowledged as the gold standard for the assessment of medical interventions [14-16] and therefore the number of RCTs is hopefully to improve in the future in burn surgery, too [17].

As far as the minimum requirements for future conference abstracts are concerned, it is rather difficult to propose detailed suggestions. Following the CONSORT and STROBE criteria it would be crucial in our view to clearly note the study design, details on recruitment of participants, study size randomization, quantitative variables, main outcome, harms and especially potential funding in the abstract form to improve reporting quality. These items would allow the abstract reader to assess the conference abstract in a more comprehensive way. However, limited available space has to be considered in this regard. Depending on conference and journal requirements word count is usually limited to 250 to 300 words for conference abstracts. Therefore the abstract format is often times strictly controlled with background, methods, results, and conclusions. Thus, investigators are influenced and guided by these requirements. In addition, the CONSORT group has calculated that within 250 to 300 words, all 17 items of the CONSORT abstract recommendations are met.

In several studies, it was reported that the information given for trials in conference proceedings is far from being optimal [18,19]. Clear, transparent and accurate reporting or research is important because it enables readers to understand what was done and to acess the applicability and relevance of the findings. The extension of CONSORT aims to improve the reporting in randomized-control trials. However, we believe, beyond the primary scope of CONSORT for RCT and STROBE for observational studies, that in general, the abstracts reporting quality will benefit from the implementation of standardized criteria to improve the reporting quality, both in the abstract as well as in a potential subsequent full manuscript.

### Limitations

Several limitations of our study should be noted. First, we evaluated the quality of reporting, which is not the same as the methodologic quality of the study. It is possible that a poorly reported study is well designed and executed, and a well-reported one may have several shortcomings. However, empirical evidence exists that indicates poorly reported studies are associated with larger estimates of intervention effect, i.e. poor reporting reflects poor methodology, which in turn is associated with biased results [20].

Second, given the suggestions of the conference organizing committee regarding the abstract format and the restricted word count, it has to take into account that the implementation of a more comprehensive reporting in abstracts might be hampered by the abstract requirements stated by the conference organizers. Also, submission and selection process of journals, including author request of presentation (oral, poster) could influence the type of presentation. And also it should be acknowledged that abstract are submitted and judged by category. Therefore request of presentation by author or categorisation by journal could be a potential confounder in this study.

Furthermore, we analysed only studies of evidence level 1++ to 2- according to the Scottish Intercollegiate Guidelines Network (SIGN). Because of the low evidence, experimental studies, new techniques and describing new concepts were excluded from the analysis. In this context, laboratory studies and animal studies were classified as experimental studies. Furthermore, it is possible that the analysed study designs in abstracts are misinterpertated. In addition, the CONSORT criteria were suggested primarily for randomized-controlled trials which took only about 2-7% of study designs in conference abstracts. We also acknowledge that STROBE is currently limited to three main observational study designs: cohort, case-control, and cross-sectional studies. Furthermore the STROBE criteria were not primary designed for abstracts. But no other better instrument was available and because of its specificity for observational studies, we think it was the best way to evaluate the reporting quality. While no such statements or checklists for experimental or other study designs were available, we applied the Timmer instrument for abstract quality in addition to the aforementioned measures. The Timmer instrument is a valid quality assessment tool for conference abstracts and comparing these three instruments for analysing the abstract quality leads to an increased internal validity and reliability of the study. In addition, it should be kept in mind that in this study misclassification in approaching to CONSORT and STROBE criteria could occur [21]. To minimize this effect, criteria for reporting in conference abstracts were analysed by two researchers independently. Finally, we compared just two different years, with two different sets of abstract. This limited amout of sampling gives some limitation in external validity of these trials. Therefore further analysis of more data, especially multiple year comparisons and comparing trends over time, is needed.

## Conclusion

Poster abstracts at the American Burn Association annual meetings 2000 and 2008 are not necessarily inferior to oral abstracts as far as study design and reporting quality of clinical trials are concerned. However, obligatory use of CONSORT and STROBE criteria in submitting process of clinical trials, such as the *Journal of Thrombosis and Haemostasisas*, as well as the comprehensive use of both criteria by authors independently, might further increase the quality of reporting ABA conference abstracts and help to facilitate peer review [22] in the future.

## Competing interests

The authors declare that they have no competing interests. No author received internal or external funding.

## Authors' contributions

KK developed the idea, analyzed all abstracts and wrote the manuscript. UY co-analyzed the data and co-wrote the manuscript. HOR provided the abstracts, co-developed the idea and reviewed the manuscript. PMV inspired the idea, co-analyzed the data and critical reviewed the manuscript. All authors read and approved the final manuscript

## Pre-publication history

The pre-publication history for this paper can be accessed here:

http://www.biomedcentral.com/1471-2288/11/161/prepub

## Supplementary Material

Additional file 1 **CONSORT-Checklist**. CONSORT-Checklist for RCT Click here for file

Additional file 2 **STROBE-Checklist**. STROBE-Checklist for observational studies Click here for file

Additional file 3 **Timmer quality scoring instrument for abstract quality**. Timmer quality scoring instrument for abstracts Click here for file
