# Supplementary material for: Quality of reporting according to the CONSORT, STROBE and Timmer instrument at the American Burn Association (ABA) annual meetings 2000 and 2008
Source: BMC Med Res Methodol. 2011 Nov 29;11:161. doi: 10.1186/1471-2288-11-161 (PMC3247193; doi:10.1186/1471-2288-11-161)
Supplement: Additional file 1 — CONSORT-Checklist. CONSORT-Checklist for RCT [file 1471-2288-11-161-S1.DOC]

Additional file 1. CONSORT-Checklist

| **Item** | **Description** |
| --- | --- |
| Title | Identification of the study as randomized |
| Authors | Contact details for the corresponding author |
| Trial design | Description of the trial design (eg. Parallel, cluster, non-inferiority) |
| **Methods** |  |
| Participants | Eligibility criteria for participants and the settings where the data were collected |
| Interventions | Interventions intended for each group |
| Objective | Specific objective or hypothesis |
| Outcome | Clearly defined primary outcome for this report |
| Randomization | How participants were allocated to interventions |
| Blinding (masking) | Whether or not participants, care givers, and those assessing the outcomes were blinded to group assignment |
| **Results** |  |
| Numbers randomized | Number of participants randomized to each group |
| Recruitment | Trial status |
| Numbers analyzed | Number of participants analyzed in each group |
| Outcome | For the primary outcome, a result for each group and the estimated effect size and its precision |
| Harms | Important adverse events or side-effects |
| Conclusions | General interpretation of the results |
| Trial registration | Registration number and name of the trial register |
| Funding | Source of funding |
