# Supplementary material for: Quality of reporting according to the CONSORT, STROBE and Timmer instrument at the American Burn Association (ABA) annual meetings 2000 and 2008
Source: BMC Med Res Methodol. 2011 Nov 29;11:161. doi: 10.1186/1471-2288-11-161 (PMC3247193; doi:10.1186/1471-2288-11-161)
Supplement: Additional file 2 — STROBE-Checklist. STROBE-Checklist for observational studies [file 1471-2288-11-161-S2.DOC]

Additional file 1. STROBE-Checklist

| Title and Abstract | (a) Indicate the study’s design with a commonly used term in the title or the abstract  (b) Provide in the abstract, an informative and balanced summary of what was done and what was found |
| --- | --- |
| **Introduction** |  |
| Background/rationale | Explain the scientific background and rationale for the investigation being reported |
| Objectives | State specific objectives, including any prespecified hypotheses |
| **Methods** |  |
| Study design | Present key elements of study design early in the paper |
| Setting | Describe the setting, locations, and relevant dates, including periods of recruitment, exposure, follow up, and data collection |
| Participants | (a) Cohort study-Give the eligibility criteria, and the sources and methods of selection of participants. Describe methods of follow up  Case-control study-Give the eligibility criteria, and the sources and methods of case ascertainment and control selection. Give the rationale for the choice of cases and controls  Cross-sectional study-Give the eligibility criteria, and the sources and methods of selection of participants  (b) Cohort study-For matched studies, give matching criteria and number of exposed and unexposed  Case-control study-For matched studies, give matching criteria and the number of controls per case |
| Variables | Clearly define all outcomes, exposures, predictors, potential confounders, and effect modifiers. Give diagnostic criteria, if applicable |
| Data sources/ measurement | For each variable of interest, give sources of data and details of methods of assessment (measurement). Describe comparability of assessment methods if there is more than one group |
| Bias | Describe any efforts to address potential sources of bias |
| Study size | Explain how the study size was arrived at |
| Quantitative variables | Explain how quantitative variables were handled in the analyses. If applicable, describe which groupings were chosen, and why |
| Statistical methods | (a) Describe all statistical methods, including those used to control for confounding  (b) Describe any methods used to examine subgroups and interactions  (c) Explain how missing data were addressed  (d) Cohort study. If applicable, explain how loss to follow up was addressed  Case-control study-If applicable, explain how matching of cases and controls was addressed  Cross-sectional study-If applicable, describe analytical methods taking account of sampling strategy  (e) Describe any sensitivity analyses |
| **Results** |  |
| Participants | (a) Report the numbers of individuals at each stage of the study, e.g., numbers potentially eligible, examined for eligibility, confirmed eligible, included in the study, completing follow up, and analyzed  (b) Give reasons for nonparticipation at each stage  (c) Consider use of a flow diagram |
| Descriptive data | (a) Give characteristics of study participants (e.g., demographic, clinical, social) and information on exposures and potential confounders  (b) Indicate the number of participants with missing data for each variable of interest  (c) Cohort study-Summarize follow-up time (e.g., average and total amount) |
| Outcome data | Cohort study-Report numbers of outcome events or summary measures over time  Case-control study-Report numbers in each exposure category, or summary measures of exposure  Cross-sectional study-Report numbers of outcome events or summary measures |
| Main results | (a) Give unadjusted estimates and, if applicable, confounder-adjusted estimates and their precision (e.g., 95% confidence interval). Make clear which confounders were adjusted for and why they were included  (b) Report category boundaries when continuous variables were categorized  (c) If relevant, consider translating estimates of relative risk into absolute risk for a meaningful time period |
| Other analyses | Report other analyses done-e.g., analyses of subgroups and interactions, and sensitivity analyses |
| **Discussion** |  |
| Key results | Summarize key results with reference to study objectives |
| Limitations | Discuss limitations of the study, taking into account sources of potential bias or imprecision.  Discuss both direction and magnitude of any potential bias |
| Interpretation | Give a cautious overall interpretation of results considering objectives, limitations, multiplicity of analyses, results from similar studies, and other relevant evidence |
| Generalizability | Discuss the generalizability (external validity) of the study results |
| **Other information** |  |
| Funding | Give the source of funding and the role of the funders for the present study and, if applicable, for the original study on which the present article is based |
