# Supplementary material for: Quality of reporting according to the CONSORT, STROBE and Timmer instrument at the American Burn Association (ABA) annual meetings 2000 and 2008
Source: BMC Med Res Methodol. 2011 Nov 29;11:161. doi: 10.1186/1471-2288-11-161 (PMC3247193; doi:10.1186/1471-2288-11-161)
Supplement: Additional file 3 — Timmer quality scoring instrument for abstract quality. Timmer quality scoring instrument for abstracts [file 1471-2288-11-161-S3.DOC]

Additional file 1. Timmer quality scoring instrument for abstract quality.

| **Quality assessment** | yes | partial | no | n/a |
| --- | --- | --- | --- | --- |
| 1. Question / objective sufficiently described? |  |  |  |  |
| 1. Design evident and appropriate to answer study question? |  |  |  |  |
| 1. Subject characteristics sufficiently described? |  |  |  |  |
| 1. Subjects appropriate to the study question? |  |  |  |  |
| 1. Controls used and appropriate? **(if no control, check no)** |  |  |  |  |
| 1. Method of subject selection described and appropriate? |  |  |  |  |
| 1. If random allocation to treatment groups was possible, is it described? (if not possible, check n/a) |  |  |  |  |
| 1. If blinding of investigators to intervention was possible, is it reported? (If not possible, n/a) |  |  |  |  |
| 1. If blinding of subjects to intervention was possible, is it reported? (If not possible, n/a)^1^ |  |  |  |  |
| 1. Outcome measure well defined and robust to measurement bias? Means of assessment reported? |  |  |  |  |
| 1. Confounding accounted for? |  |  |  |  |
| 1. Sample size adequate? |  |  |  |  |
| 1. Post hoc power calculations or confidence intervals reported for statistically non significant results? |  |  |  |  |
| 1. Statistical analyses appropriate? |  |  |  |  |
| 1. Statistical tests stated? |  |  |  |  |
| 1. Exact p-values or confidence intervals stated? |  |  |  |  |
| 1. Attrition of subjects and reason for attrition recorded? |  |  |  |  |
| 1. Results reported in sufficient detail? |  |  |  |  |
| 1. Do the results support the conclusions? |  |  |  |  |
| Sum (items 1-19) |  |  |  |  |

**Scoring:** For each applicable item, 0-1 points are awarded (1 if met, 0 if not met). For each item which is not applicable, such as blinding of subjects in observational tirals, items are subtracted from the total possible score. The summary score is calculated by summate the score of each item.
